# User-Centered Framework for Implementation of Technology (UFIT): Development of an Integrated Framework for Designing Clinical Decision Support Tools Packaged With Tailored Implementation Strategies

**DOI:** 10.2196/51952

**Published:** 2024-05-21

**Authors:** Jessica Ray, Emily Benjamin Finn, Hollyce Tyrrell, Carlin F Aloe, Eliana M Perrin, Charles T Wood, Dean S Miner, Randall Grout, Jeremy J Michel, Laura J Damschroder, Mona Sharifi

**Affiliations:** 1 Department of Health Outcomes and Biomedical Informatics University of Florida College of Medicine Gainesville, FL United States; 2 Department of Pediatrics Yale University School of Medicine New Haven, CT United States; 3 Academic Pediatric Association McLean, VA United States; 4 Department of Pediatrics Johns Hopkins University School of Medicine and School of Nursing Baltimore, MD United States; 5 Department of Pediatrics Duke University School of Medicine Durham, NC United States; 6 Departments of Pediatrics and Internal Medicine East Carolina University Greenville, NC United States; 7 Department of Pediatrics Indiana University School of Medicine and Regenstrief Institute Indianapolis, IN United States; 8 Department of Pediatrics Perelman School of Medicine University of Pennsylvania Philadelphia, PA United States; 9 Implementation Pathways LLC and Veterans Affairs Center for Clinical Management Research Ann Arbor, MI United States

**Keywords:** user-centered design, implementation science, clinical decision support, human factors, implementation, decision support, develop, development, framework, frameworks, design, user-centered, digital health, health technology, health technologies, need, needs, tailor, tailoring, guidance, guideline, guidelines, pediatric, pediatrics, child, children, obese, obesity, weight, overweight, primary care

## Abstract

**Background:**

Electronic health record–based clinical decision support (CDS) tools can facilitate the adoption of evidence into practice. Yet, the impact of CDS beyond single-site implementation is often limited by dissemination and implementation barriers related to site- and user-specific variation in workflows and behaviors. The translation of evidence-based CDS from initial development to implementation in heterogeneous environments requires a framework that assures careful balancing of fidelity to core functional elements with adaptations to ensure compatibility with new contexts.

**Objective:**

This study aims to develop and apply a framework to guide tailoring and implementing CDS across diverse clinical settings.

**Methods:**

In preparation for a multisite trial implementing CDS for pediatric overweight or obesity in primary care, we developed the User-Centered Framework for Implementation of Technology (UFIT), a framework that integrates principles from user-centered design (UCD), human factors/ergonomics theories, and implementation science to guide both CDS adaptation and tailoring of related implementation strategies. Our transdisciplinary study team conducted semistructured interviews with pediatric primary care clinicians and a diverse group of stakeholders from 3 health systems in the northeastern, midwestern, and southeastern United States to inform and apply the framework for our formative evaluation.

**Results:**

We conducted 41 qualitative interviews with primary care clinicians (n=21) and other stakeholders (n=20). Our workflow analysis found 3 primary ways in which clinicians interact with the electronic health record during primary care well-child visits identifying opportunities for decision support. Additionally, we identified differences in practice patterns across contexts necessitating a multiprong design approach to support a variety of workflows, user needs, preferences, and implementation strategies.

**Conclusions:**

UFIT integrates theories and guidance from UCD, human factors/ergonomics, and implementation science to promote fit with local contexts for optimal outcomes. The components of UFIT were used to guide the development of Improving Pediatric Obesity Practice Using Prompts, an integrated package comprising CDS for obesity or overweight treatment with tailored implementation strategies.

**Trial Registration:**

ClinicalTrials.gov NCT05627011; https://clinicaltrials.gov/study/NCT05627011

## Introduction

Electronic health record (EHR)–based clinical decision support (CDS) tools can accelerate clinicians’ adoption of evidence-based practice [1,2]. However, the potential for a broad and sustained impact on care delivery is limited by institutional policies, EHR system constraints, and end user behaviors that can inhibit dissemination and implementation across heterogeneous settings [1-4]. Failing to situate CDS tools within existing workflows or overlooking human and system-level barriers or facilitators can produce unintended consequences. Poor EHR usability has been associated with clinician burnout, alert fatigue, and inappropriate care, suggesting potentially harmful effects of mismatches between technology, site-specific context, and user needs [5-7]. Successful dissemination and implementation across diverse clinical contexts require balancing the fidelity of core functions with adaptation to ensure compatibility with shared and divergent user needs and contexts [8].

User-centered design (UCD) offers a structured, iterative process to refine an intervention by identifying and meeting individual user needs and preferences at the outset and throughout design and development. This approach centers on users, clinical context, and user feedback throughout design and development. Well-executed UCD can elicit and address critical information about user cognition, affect, and work systems [9,10]. Although extant technology standards explicitly call for UCD, these processes are applied inconsistently during EHR tool development due to variability in technology developers’ UCD knowledge and resources [10]. UCD embraces a variety of user engagement methods to distill and clarify the steps needed to optimally design systems for users. However, UCD models are designed to provide a process for design instead of theory-based approaches to guide the exploration of use context. Furthermore, UCD is often more focused on technology development than on strategies to support its adoption.

To facilitate adoption, the fields of implementation science and human factors/ergonomics (HFE) offer approaches for understanding important mediators and modifiers of the implementation of interventions like CDS. By providing theoretical and empirical insights into human-system interactions and cognitive processes, findings from these fields support design. For example, insights can be used to identify and tailor effective implementation strategies (eg, trainings, audits, and feedback) and have been bolstered recently through the application of the UCD process to design implementation strategies. In this study, we developed and applied an integrated framework, the User-Centered Framework for Implementation of Technology (UFIT), in preparation for a multisite clinical effectiveness-implementation randomized controlled trial, Improving Pediatric Obesity Practice Using Prompts (iPOP-UP; ClinicalTrials.gov NCT05627011) to improve guideline-concordant care for children with overweight or obesity in pediatric primary care. UFIT builds upon the UCD process model by layering HFE theories to ground our understanding of user-system interactions within the clinical setting and an implementation science framework to capture aspects of the broader context, informing implementation and sustained use of our CDS intervention.

## Methods

### Conceptual Foundations

**Figure 1:**
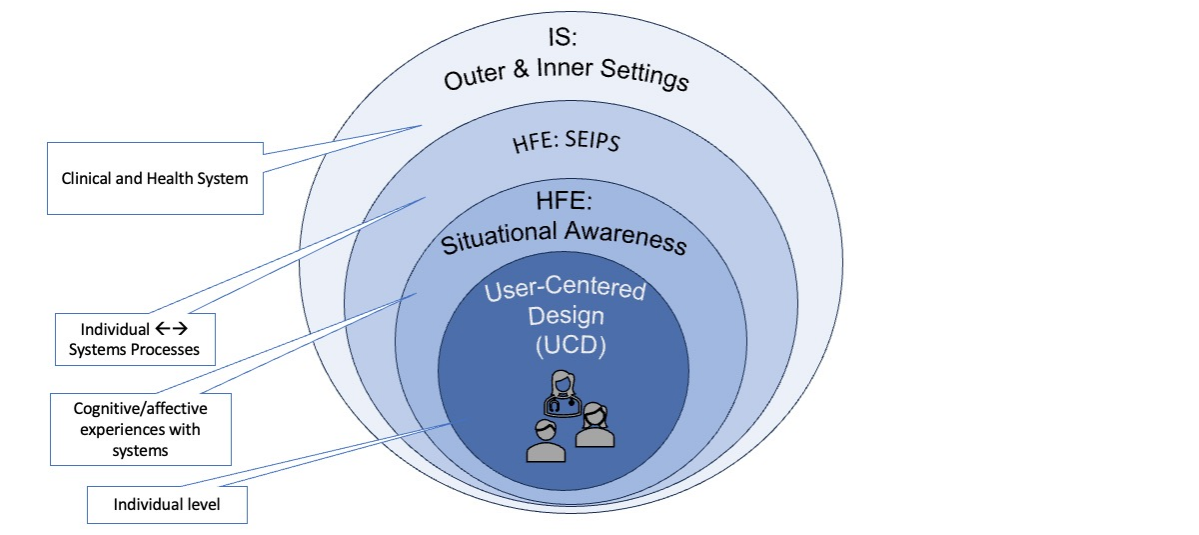
Conceptual integration of models and theories. HFE: human factors/ergonomics; IS: implementation science; SEIPS: Systems Engineering for Patient Safety; UCD: user-centered design.

We first developed a foundational conceptual framework to guide the development of the UFIT process model. As shown in Figure 1, we draw on multiple well-established frameworks and theoretical domains. We started by centering concepts from typical UCD approaches, placing the user at the center throughout design and development. Next, we link key HFE theoretical models (eg, Situation Awareness and Systems Engineering Initiative for Patient Safety) and 1 implementation science framework, the Consolidated Framework for Implementation Research (CFIR), to expand the understanding of factors that may help or hinder design or implementation of interventions [11-14].

Incorporating HFE extends UCD’s focus on the user by adding deeper consideration of the user’s interactions across the broader clinical system and the local sociotechnical environment. Situation awareness theory expands our inquiry into human-system interactions by identifying the ways in which clinicians gather, process, and comprehend information in the clinical setting to predict future states and to inform their decision-making [11,15]. Health care environments are complex, where interactions occur among and between people engaged in the system (eg, clinician, patient, and chief medical information officer) as well as tools and technologies needed to complete tasks within the clinical environments [12,16]. Implementing a change in any one component of this type of complex sociotechnical environment can create unintended consequences. We used these insights to guide CDS design requirements to optimize human-system interactions that supported decision-making for best outcomes.

While prior work offers insight into features that can support the adoption of CDS, only recently have researchers sought to identify the implementation features that maximize the adoption (use) of CDS [17]. This recent work provides a foundation for understanding feature selection and paired implementation strategies that have been shown effective, yet it still leaves the important design considerations of how these strategies should be tailored to support local contexts. To tailor implementation strategies, we incorporated the CFIR, a “meta-theoretical” framework with well-defined constructs (ie, potential barriers and facilitators to effective implementation) grouped across five domains: (1) innovation characteristics, (2) outer setting, (3) inner setting, (4) the roles and characteristics of the individuals involved, and (5) implementation processes [13,18]. The CFIR provides a guide to systematically assess how each of these 5 domains may influence implementation. Incorporating components of the CFIR helped to inform understanding of multilayered complexities that influence end users’ decisions to adopt CDS tools into their workflow. For example, we sought to understand not only clinicians’ current and desired behaviors of interest (eg, “Individual Capability” and “Motivation”) but also influences from multiple levels of the context within which clinicians act (eg, “Relative Priority” within the “Inner Setting” that may include the clinic) and (eg, “Local Conditions” within the “Outer Setting” that may include the broader health system*)*. Incorporating the CFIR into UFIT helps to inform our understanding of the diverse needs and constraints of implementation sites more clearly than if we only used UCD, which does not explicitly cover these considerations.

### UFIT Development and Application

**Figure 2:**
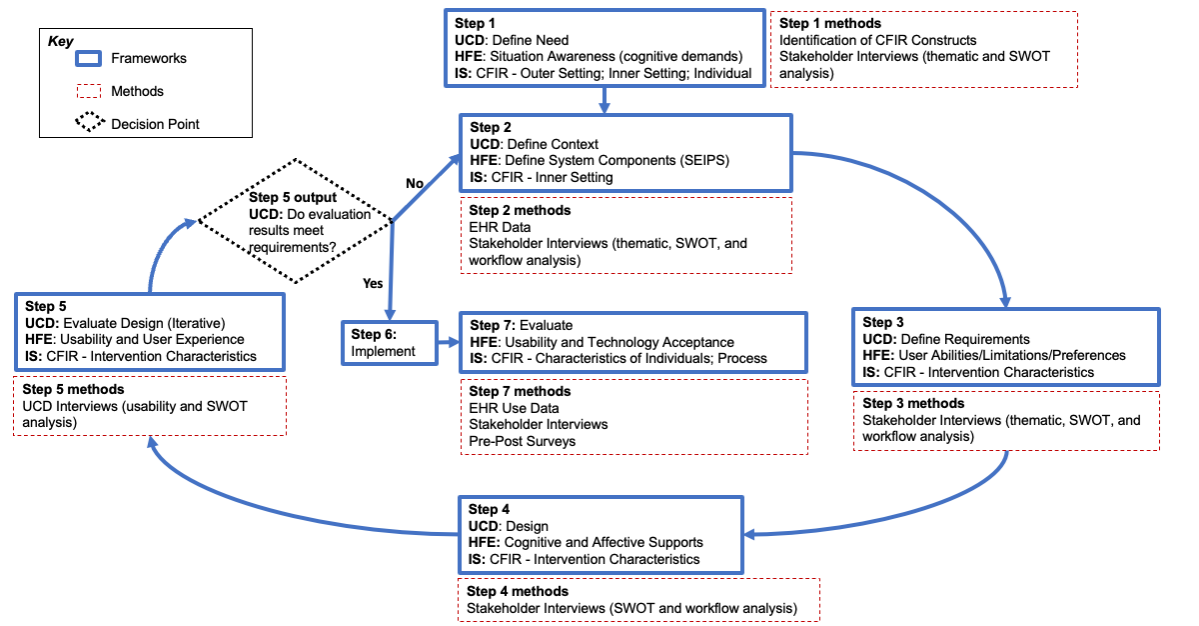
The User-Centered Framework for Implementation of Technology (UFIT): steps and methods for evaluation. CFIR: Consolidation Framework for Implementation Research; EHR: electronic health record; HFE: human factors/ergonomics; IS: implementation science; SEIPS: Systems Engineering for Patient Safety; SWOT: strengths, weaknesses, opportunities, and threats; UCD: user-centered design.

Based on concepts from our foundational framework, we developed steps for designing an EHR-based CDS tool with companion implementation strategies that are tailored to the needs of multiple heterogeneous pediatric primary care clinics. The resulting 7-step framework, the User-Centered Framework for Implementation of Technology (UFIT), is presented in Figure 2. Concepts from our foundational framework (see Figure 1) are identified for each of the 7 steps. UFIT provides structured guidance for key actions and topics of inquiry for each step. UFIT helps to identify idiosyncratic workflows and site-specific processes to inform the design of a technology-based intervention with tailored implementation strategies.

We applied each step in UFIT to prepare for the iPOP-UP randomized controlled trial that aims to improve guideline-concordant care for children with overweight or obesity in pediatric primary care. iPOP-UP packages a clinical CDS with implementation strategies into a single bundle.

### Preparation

Methods used to apply UFIT within our formative evaluation included qualitative and quantitative methods such as semistructured interviews with clinicians and staff, EHR data queries, and surveys (Table 1). Participants and data collection and analyses used throughout the process are detailed in Table 1. Institutional review boards at each site reviewed and determined the protocol for these data collections as exempt.

In preparation for engaging stakeholders in UFIT, 9 experts from our core transdisciplinary study team, with expertise in pediatric obesity, primary care practice patterns, health inequities, implementation science, and clinical informatics, weighted their perceived relevance of each of the original CFIR’s 39 constructs to evaluating determinants of implementation of clinical practice guidelines and of adoption of CDS in pediatric primary care. This weighting was followed by a group discussion to achieve consensus; 26 constructs were identified as highly relevant priorities to inform the construction of the interview guides and survey used in our formative evaluation. While we used the original version of the CFIR to guide data collection, we mapped our findings and report based on the updated framework, published after our data collection was complete [9,10].

**Table 1:** Data collection participants, methods, objectives, and analyses within the User-Centered Framework for Implementation of Technology.

Objective, UFIT^a^ steps, and participants				Methodology details				
				Method		Objectives		Analysis
**Explore needs and context**								
	**1**							
		Patients aged 2-18 years with BMI ≥85th percentile	EHR^b^ data query		—^c^		Descriptive analysis to analyze patient demographics and EHR documentation of key clinician behaviors related to overweight or obesity management	
	**1, 2, 3**							
		Level 1 stakeholders (anticipated end users)^d^	Semistructured interview		Explore clinicians’ goals, experiences, workflows, and expectations in addressing overweight and obesity in primary care Explore perceptions of what changes are needed and possible solutions, including those beyond the constraints of current EHR systems		Rapid qualitative analysis Content analysis to code workflow data, graphically represented in Sankey diagrams SWOT^e^ analyses	
		Level 2 stakeholders (non-PCC^f^ clinicians, staff who will be impacted by or influence implementation)^g^	Semistructured interview		Explore clinicians’ goals, experiences, workflows, and expectations in addressing overweight and obesity in primary care Explore perceptions of what changes are needed and possible solutions, including those beyond the constraints of current EHR systems		SWOT analysis	
**Iterative design**								
	**4, 5**							
		Level 1 stakeholders (anticipated end users)^d^	Focus group		Confirm and refine workflow patterns Gather feedback on initial design prototypes		Field notes analyzed through summarization feedback for each CDS^h^ tool presented. Compiled data reviewed against prior findings, requirements, and workflow patterns to determine if additional modifications were needed.	
		Level 1 stakeholders (anticipated end users)^d^	Semistructured interview		Confirm and refine workflow patterns Gather feedback on initial design prototypes		—	
		Nursing and medical assistant staff	Semistructured interview		Confirm and refine workflow patterns Gather feedback on initial design prototypes		Field notes were analyzed through summarization feedback for each CDS tool presented. Compiled data were reviewed against prior findings, requirements, and workflow patterns to determine if additional design modifications were needed.	
**Evaluate trial impact**								
	**7**							
		Level 1 stakeholders (anticipated end users)^d^	Semistructured interview		Gather feedback on intervention and implementation strategies		Thematic analysis	
		Level 1 stakeholders (anticipated end users)^d^	Postsurvey		Gather feedback on intervention and implementation strategies		Descriptive analysis Wilcoxon sum χ2 tests	
		Patients aged 2-18 years with BMI ≥85th percentile	EHR data query		Gather feedback on intervention and implementation strategies		Descriptive analysis to analyze patient demographics and EHR documentation of key clinician behaviors related to overweight or obesity management	

^a^UFIT: User-Centered Framework for Implementation of Technology.

^b^EHR: electronic health record.

^c^Not available.

^d^Primary care clinicians included physicians with training in General Pediatrics, Internal Medicine-Pediatrics (Med-Peds), and Family Medicine; nurse practitioners; and physician assistants.

^e^SWOT: strengths, weaknesses, opportunities, and threats.

^f^PCC: primary care clinician.

^g^Level 2 stakeholders included Dietitians and healthy weight program clinicians and staff, informatics professionals, clinical site practice managers, and quality, safety, and human factors professionals.

^h^CDS: clinical decision support.

### Defining Need, Context, and Requirements (UFIT Steps 1-3)

To understand local uptake of clinical practice guidelines and expert recommendations at primary care clinics, we first quantified clinicians’ documented care for children with overweight or obesity through EHR data queried from 84 pediatric primary care practices affiliated with the 3 participating health systems all using Epic EHR systems. Together with primary care physicians with training in general pediatrics and medicine-pediatrics and expertise in clinical informatics from each site, we developed a data query protocol for all well-child visits for children aged 2-12 years with overweight or obesity, defined as a BMI at or above the 85th percentile for age and sex. From these data, we used descriptive analyses to summarize patient demographics and documentation of key clinician behaviors related to overweight or obesity management to define gaps and variability in guideline-concordant care within each health system.

**Figure 3:**
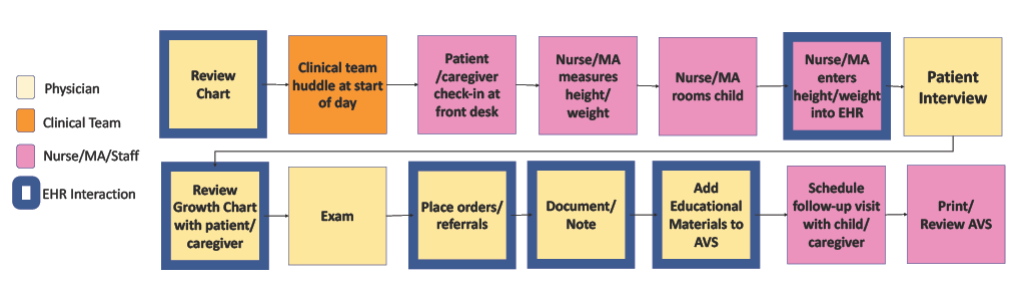
Example workflow mapping during data collection. AVS: after visit summary; EHR: electronic health record; MA: medical assistant.

**Figure 4:**
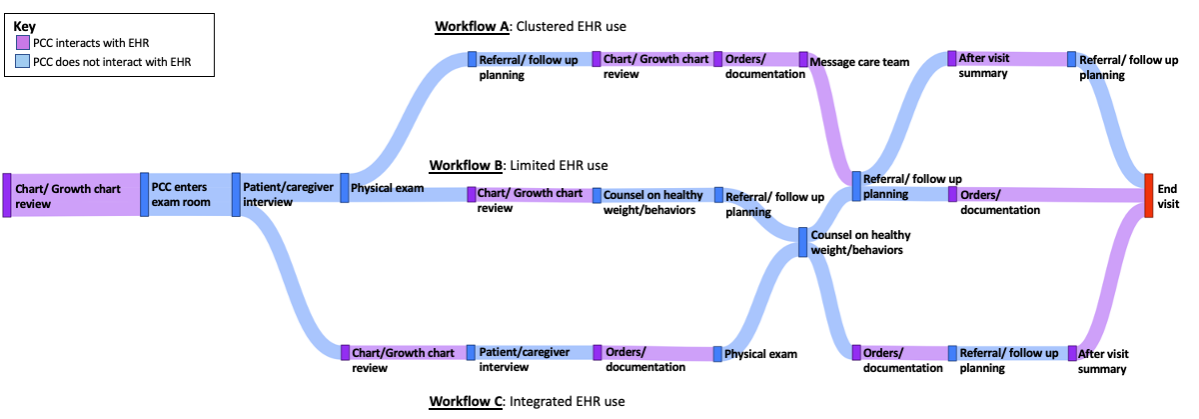
Sankey diagram of clinician workflow and EHR use patterns during pediatric primary care visit. EHR: electronic health record; PCC: primary care clinician.

Next, we sought to qualitatively understand primary care clinicians’ (PCCs’) behaviors and the cognitive and affective factors driving these behaviors. We developed semistructured interview guides to elicit cognitive features supporting clinicians’ situation awareness [19]. To understand context, we applied an adapted HFE approach by having PCC stakeholders map their workflows during a well-child visit for children with overweight or obesity during the first round of interviews (Figures 3 and 4). To map the sociotechnical system, researchers often conduct observations capturing characteristics of system components and interactions among these components. For example, a clinician might interact with a patient in an exam room through a series of questions, then document the answers in the EHR, following local workflow structures and meeting external regulations and billing requirements. Given the broad range of systems and their geographic diversity, as well as the timing coinciding with the COVID-19 pandemic, we adapted the approach by conducting cognitive walkthroughs with a graphical sticky note exercise to map the system features and clinician workflow during our interviews. Based on our selected CFIR constructs, interview questions also explored prior experiences with CDS implementations in the participating organizations, clinician capability and motivation in managing overweight or obesity, and their agreement with associated guidelines. These questions encouraged “Reflecting and Evaluating,” a key CFIR construct. The team also noted that CFIR’s “Innovation Design” and “Tailoring Strategies” constructs are strengthened via UCD. A structured approach to CDS design and tailoring of implementation strategies within these contexts can optimize the overall performance and users’ experiences while minimizing the potential for unintended consequences.

To achieve this, we purposively recruited 2 groups of key informants (clinician end users and non-PCC clinicians and staff who will be impacted by or influence implementation), defined by their proximity to using the CDS, (Table 1) for semistructured interviews to define the need, context, and requirements (UFIT steps 1, 2, and 3) at each site. Interviews lasted approximately 45 minutes. The PCC stakeholder interview guide was designed to produce four distinct categories of data: (1) barriers and facilitators of care for pediatric overweight or obesity, (2) workflow, (3) barriers and facilitators to EHR tool implementation and use, and (4) PCC needs and possible solutions to improve care for patients with overweight or obesity. Analysis for each of these sections included (1) rapid identification of themes for barriers and facilitators of care; (2) Sankey diagrams of clinician workflows; (3) strengths, weaknesses, opportunities, and threats analysis to understand barriers and facilitators to EHR tool implementation; and (4) rapid qualitative analysis, identifying new tools PCCs would find helpful and the potential users of those tools. Interviews were professionally transcribed and independently coded by at least 2 researchers using a rapid assessment procedure [11]. The 3-person interdisciplinary coding group with experience in human factors, public health, and pediatric health care research initially identified broad categories to define barriers and facilitators (eg, patient or family factors and clinician-level factors). We then defined subcategories and iteratively refined them as transcripts were coded and themes emerged within the barriers and facilitators. Coders summarized transcript segments and noted line numbers within each category or subcategory. Strengths, weaknesses, opportunities, and threats analyses were conducted using PCC interview transcripts or real-time field notes for additional stakeholder interviews. A content analysis of the steps clinicians took prior to and during a patient visit was completed to understand workflows and summarized using a Sankey diagram (Figure 3) to create a visualization of the findings from the content analysis.

To define design requirements (step 3), we mapped CDS components to initial interview findings including positive and negative experiences with EHR tools, perceptions of existing CDS tools, and desired CDS tools. We engaged our transdisciplinary study team to narrow the requirements generated from PCC interviews. Investigators from each site were asked to document existing tools that shared core functional components of the CDS to identify the baseline state and past experiences with EHR design in their local environments. The Sankey diagrams were then used to identify timing requirements for CDS tools within clinician workflows. In alignment with HFE, we used UFIT to outline user workload and competing priorities (eg, the need for CDS to facilitate the steps of a clinician’s work in a 20-minute visit). To define initial design requirements for our implementation strategies, we linked potential barriers and threats to adoption using the Expert Recommendations for Implementing Change taxonomy of implementation strategies [12]. From CFIR, we consider the CDS characteristics and associated implementation strategies (eg, use “relative advantage” to ensure completing care tasks, such as ordering labs based on new guidelines vs ordering labs saved as favorites that may not be in line with new guidelines).

### Evaluating Design (UFIT Steps 4 and 5)

We translated data from step 3 into an initial set of design prototypes that we presented to our transdisciplinary study team to identify opportunities for further refinement and potential local EHR system constraints. Data from site-specific focus groups was used to confirm and refine workflow patterns. Next, we convened focus groups of PCCs in a first round of design and evaluation (steps 4 and 5) to elicit feedback on early design ideas and confirm workflow patterns. These sessions also allowed us to gather feedback on initial design prototypes and facilitate consensus building within each site to allow us to narrow design plans for subsequent rounds of user testing. The evaluation was informed by the HFE concepts of “usability” and “user experience” as well as key components of the “intervention characteristics” as detailed in CFIR [13,14]. Iterative rounds of design and testing (steps 4 and 5) consisted of individual interviews using images of refined CDS tools in a test environment applied to a hypothetical patient. Between our first and second rounds, we transitioned our prototypes from wireframe to the EHR test environment, from which we captured photos and videos. Beginning with our second round of evaluation, we conducted individual interviews with PCCs using a think-aloud interview guide based on EHR photos and videos to understand user preferences for the tools. Participants were prompted for summative feedback at the end of each session to elicit ideas on both technology design and potential implementation strategies to support adoption. Following each iteration, evaluation feedback was compared with data gathered in initial PCC interviews to confirm that our design was meeting user needs (step 1) and context (step 2) and to assess for any further refinement to our definitions of the context (step 2) and user requirements (step 3). While early rounds focused largely on CDS design testing, later rounds introduced potential implementation strategies to assess suitability. This iterative process was repeated based on meetings of the development team until all feasible user requirements were addressed. Drawing from the recent CFIR 2.0 Outcomes Addendum, we recognize that while formative evaluation may allow us to anticipate outcomes of an intervention and its implementation, only through summative evaluation of the implementation package will we be able to understand its actual impact [15].

## Results

### Defining Need, Context, and Requirements (UFIT Steps 1-3)

EHR data analysis demonstrated gaps in documented evidence of diagnosis and counseling. Specifically, among children aged 2-12 years with overweight or obesity, 57.3% (n=73,586) had any visit or problem list diagnosis reflecting elevated BMI; this ranged from a low of 18.7% (n=11,073) at the study site with the lowest such documentation to 71.2% (n=51,993) at the site with the highest. In addition, only 0.3% (n=73,586) of visits with such patients included documented nutrition or physical activity counseling.

We conducted qualitative interviews with 21 PCCs’ end users and 20 Level 2 stakeholders who would potentially be impacted or involved with the CDS and implementation but were not direct end users. Qualitative analysis of PCC interviews elicited clinicians’ goals, experiences, and expectations in addressing overweight or obesity in primary care. This included mapping both cognitive and affective dimensions driving behavior to better understand the need for decision support. PCC interviews demonstrated how clinicians encourage families to take the lead in conversations and ask permission to discuss weight, but fear of causing harm via weight stigma (clinician affect) is a barrier for some PCCs, leading to hesitancy about these conversations. Data from both PCC and additional stakeholder interviews further identified barriers and facilitators associated with CDS implementation. For example, participants spoke about prior implementations of EHR technologies and the types of implementation strategies (eg, live training sessions vs prerecorded webinars vs handouts) users in each setting preferred and why (eg, information being available when they needed it). Knowledge of these barriers provided us with targeted guidance on how, when, and where in the decision-making process to offer decision support.

To define context, workflow data captured in PCC interviews (1) provided an understanding of contextual facilitators and barriers to care, (2) identified key interactions with technology within the care context, and (3) mapped variation in workflows and EHR interactions. These data showed 3 general patterns of EHR use during a patient visit (Figure 4). The first pattern was characterized by users clustering their interactions with the EHR in the middle of the visit. Users from the second pattern described very limited use of the EHR while in the room with the patient and family, often only when showing the growth chart and placing orders at the end of a visit. The final pattern showed repeated interactions with the EHR integrated throughout the visit. These features provided the foundation for designing our CDS and implementation package to meet the needs of different sociotechnical systems and workflows. For example, clinics with colocated dietitians might offer nutritional counseling with a registered dietitian during a routine office visit, a very different workflow from clinics without this specialized support onsite.

To comprehensively characterize the context for the implementation of our CDS package, we examined how care for children with overweight or obesity varied across implementation sites. PCC interview data also defined different prioritized barriers across the CFIR domains of inner and outer settings specific to the patient and caregiver, the clinician and care team, and the health system. For example, different challenges were faced by patients in rural versus urban study sites, which might influence a PCC’s management options or decisions. While there were commonalities expressed across settings, such as a safe place for exercise, the solutions to these challenges varied. For example, in urban settings, indoor activities might be recommended whereas in more rural settings there was often a focus on the types of outdoor activities. Barriers and facilitators rooted within the inner (eg, clinic) and outer setting (eg, health system) were considered important potential drivers influencing implementation, informing the types of implementation strategies that would support these different contexts.

To define requirements, we examined each core functional component of the original trial components alongside data collected during our initial interviews pertaining to past experiences with EHR tools and implementation experiences. We considered how and when each design component would be used in the workflow as well as the anticipated impact on clinician situation awareness and workload. For example, while we anticipated labs would routinely be ordered near the middle to end of a visit, some clinic workflows incorporated early lab ordering to facilitate the information needs of onsite clinical staff who complete lab draws during the visit. Examining design requirements for implementation, we mapped interview findings to known strategies for implementation. For example, the implementation strategies of “identifying and preparing champion” and “conducting educational outreach” were suggested by several stakeholders as important for creating buy-in and supporting clinicians’ adoption of the CDS [12,16].

### Evaluating Design (UFIT Steps 4 and 5)

Early design sessions identified the need for multiple options for accessing support including a best practice advisory, health maintenance alerts, order sets, and support embedded in a standardized note template. Based on user feedback, health maintenance alerts were later removed from the design. Potential training methods were added in later rounds of iterative design and evaluation to elicit feedback on the types of implementation strategies that should be paired with the specific tools being designed and tested. Feedback on implementation strategies supported the need for multiple training strategies while largely shifting design focus away from audit and feedback strategies.

## Discussion

### Principal Findings

The design and development of iPOP-UP provided a concrete application of the UFIT as we progressed through our formative evaluation. The UFIT sought to capture a holistic understanding of users that includes their behaviors, cognitive and affective needs, and identifying relevant multilevel barriers and facilitators that are heterogeneous across clinical contexts. Insights from our transdisciplinary study team and direct feedback from users guided our design and iterative refinement as we tailored the iPOP-UP package for each study site. This included a multipronged approach to both CDS and implementation strategy design to meet the various needs and workflows from our qualitative findings. This approach draws on the strengths of UCD, HFE, and implementation science. UCD, which often focuses on initial technology development, provides the process while HFE brings into consideration of theories of situational awareness, and cognitive and affective experiences of users interacting with systems. Finally, the CFIR, which draws from implementation science, adds consideration of even broader contextual factors critical to implementation strategy development. Together these provided the necessary framework to both adapt a CDS and tailor implementation strategies that were combined into a single bundled product: iPOP-UP. Just as UCD methods have been applied more broadly to nontechnology settings [20], though iPOP-UP development was specific to a CDS and its implementation, UFIT is likely to be useful for other evidence-based interventions as well.

### Comparison to Prior Work

While CDS tools are designed to support meaningful clinical change in local environments, maximizing adoption and scaling across heterogeneous settings presents many challenges that too often lead to failed implementation. Fitting CDS and integrated implementation strategies into local contexts are essential for high usability and sustained use, yet critical consideration of fit is often overlooked. Prior work has identified characteristics associated with successful CDS implementation [17]. Yet there still remains the need to understand how to tailor these characteristics across contexts. A user-centered, structured approach to tailoring and adapting the technology with context and tailoring implementation strategies that address user needs and navigate complex and diverse local contexts may facilitate dissemination, implementation, and sustained use of CDS. While UCD offers a proven approach to designing new technology, we incorporate HFE and implementation science frameworks into each step of this approach to more comprehensively guide the tailoring of CDS bundled implementation packages across diverse settings.

UCD is increasingly shaping intervention development and has recently been introduced within implementation science [18,21-23]. While existing frameworks, including the Accelerated Creation-to-Sustainment model, have begun to adopt iterative UCD approaches to implementation planning during the creation stage [24], our framework expands on this by providing detailed theoretical constructs to guide the simultaneous design of both technology and implementation strategies. The UFIT is a comprehensive action framework designed to simultaneously guide the design and development of CDS with implementation strategies.

### Limitations

We developed the UFIT to guide our formative evaluation; as such, this presents some limitations. First, there are numerous frameworks within both HFE and implementation science that could inform UCD. We selected broad and well-established models, frameworks, and theories for additional structure throughout the process, and we recognize some readers may feel other theories warrant inclusion within our conceptual framework to further expand the steps of UCD for CDS design, development, and implementation. Second, the depth and complexity of the theories we included may require additional understanding for designers to apply to their own work. Our intention is to provide a shared understanding of the goals and topics of inquiry at each step. Finally, while we anticipate our framework will apply beyond CDS development and to chronic diseases beyond obesity, testing in other use cases will define its boundaries of application.

### Conclusions

UFIT is a pragmatic framework expanding upon the steps of UCD by explicitly incorporating HFE and implementation science theories to holistically guide CDS design and implementation across diverse settings. It is a process framework that incorporates the assessment of determinants to inform the simultaneous design of an optimized innovation with tailored implementation strategies [25]. We demonstrate the use of UFIT to optimize the tailoring of this package to local contexts across 3 large health systems. The conceptual framework presented here was developed through transdisciplinary collaboration, and it is our hope that it will provide the structured steps necessary to guide other teams to implement integrated UCD for the design of paired CDS and implementation strategies.

## Abbreviations

CDS: clinical decision support
CFIR: Consolidated Framework for Implementation Research
EHR: electronic health record
HFE: human factors/ergonomics
iPOP-UP: Improving Pediatric Obesity Practice Using Prompts
PCC: primary care clinician
UCD: user-centered design
UFIT: User-Centered Framework for Implementation of Technology
